# Progressive Granulomatous Reaction Secondary to Permanent Polyvinyl Alcohol‐Based Dermal Filler Injection: A Report of Three Cases

**DOI:** 10.1111/jocd.70057

**Published:** 2025-02-17

**Authors:** Hang Wang, Chihchieh Lo, Dongmei Wu, He Qiu

**Affiliations:** ^1^ Department of Cosmetic and Plastic Surgery, West China Hospital of Stomatology Sichuan University Chengdu Sichuan China; ^2^ Department of Cosmetic and Plastic Surgery, West China School of Public Health and West China Fourth Hospital Sichuan University Chengdu Sichuan China

To the editor,

Long‐lasting dermal fillers are widely used in aesthetic rejuvenation due to their good biocompatibility, rarity of host immune response, and durable effects. However, the long‐term safety remains uncertain and unpredictable. Once implanted in the body, these types of fillers are recognized as exogenous substances and continue to pose varying levels of challenge to the host's immune system. The late‐onset complications associated with filler injections, mainly foreign body granuloma (FBG) reaction, seem to be more common with permanent fillers (an incidence rate of 0.2%–4%). Additionally, such granulomatous reactions they cause are usually more delayed (could up to 20 years), prolonged in course, and refractory to treatment. Granuloma formation concomitant with permanent fillers, including polymethyl methacrylate (PMMA), polyacrylamide gel, and liquid silicone, has been described but remains unknown with Polyvinyl alcohol (PVA). The existing research fails to specify whether the PVA microsphere is also a nidus or a causal trigger for the granulomatous onset. Herein, three cases are first described and checked for the clinical, immunophenotypic, and histological demonstration of the progressive granulomatous reaction induced by PVA‐based filler injection.

## 1 Cases Presentation

Written informed consent was obtained from the three patients, and the case studies were conducted according to the Declaration of Helsinki guidelines. These patients (mean age 34.33, range from 32 to 36 years) who underwent the single implantation of PVA‐based filler (Bonita, IMEIK Technology Development Co., LTD, CN) nearly 1 year prior, sought medical care for the reddish, hard nodules on the glabellar and nose regions (Table 1). Clinical examinations disclosed the development of a palpable, firm, and infiltrative mass. Repeated corticosteroid injections or lesion suction procedures were employed to manage the nodules before the visit in cases one and two. However, there was only a temporary, mild improvement in symptoms, arising with subsequent manifestations of vasodilation, skin redness, and swelling at the involved site.

**TABLE 1:** The clinical features of the three included patients.

Case	Sex	Age	Symptoms	Time to symptoms	Treatment	Outcome
Case 1	Female	35	Nose and glabellar nodules; local swelling	Postoperative one and half years	Intralesional injection of corticosteroids; surgery resection	Remission; progressive improvement
Case 2	Female	36	Nose and glabellar nodules; pressure discomfort	Postoperative 1 year	Repeated corticosteroid injections; lesion suction; surgery resection	Resolved
Case 3	Female	32	Glabellar nodules	Postoperative 11 months	Surgery resection	Resolved

**FIGURE 1:**
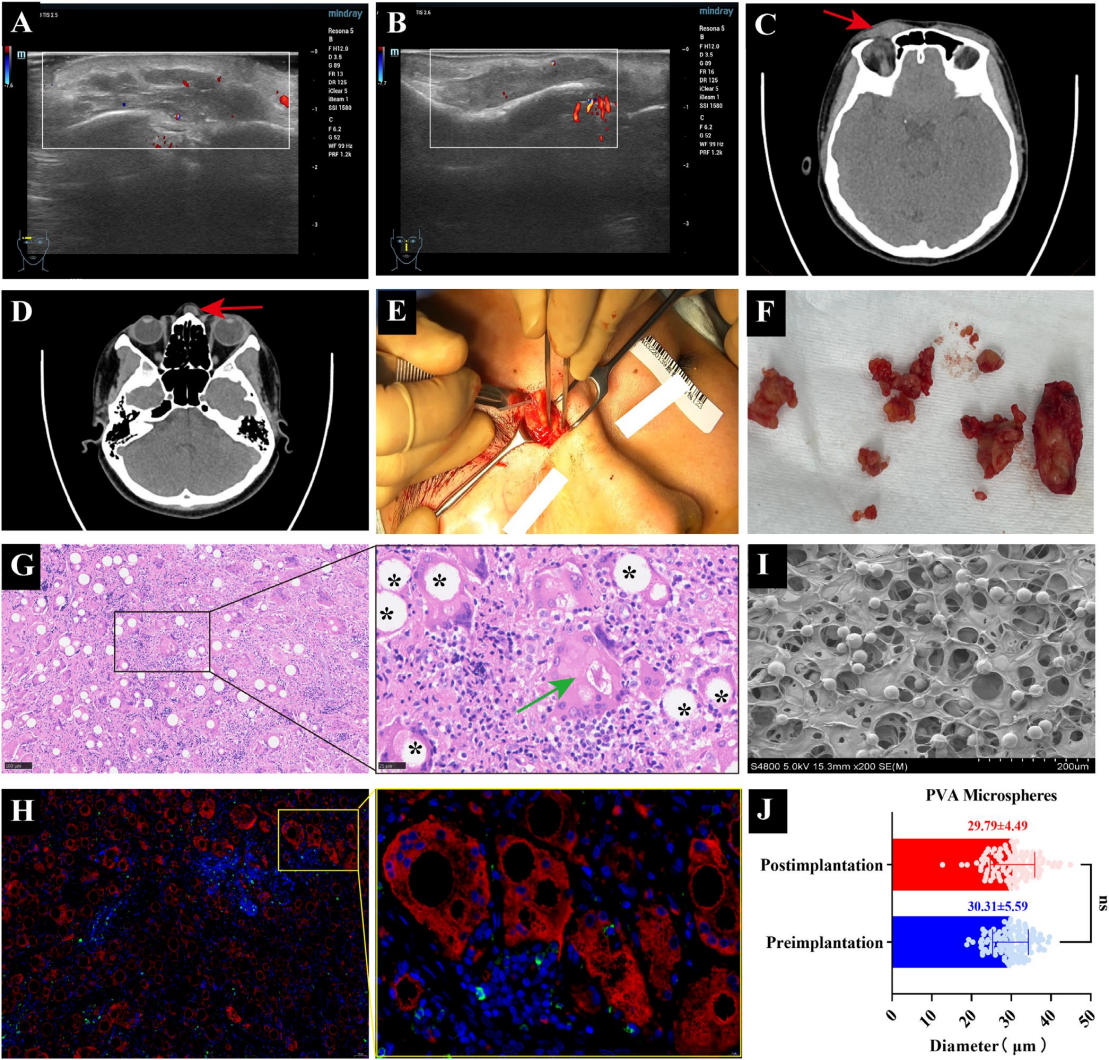
(A) Ultrasound images of the eyebrow nodule. (B) Ultrasound images of the nose nodule. (C) CT images of the eyebrow nodule. (D) CT images of the nose nodule. (E) Surgical excision of the nose masses. (F) Inflammatory masses. (G) Hematoxylin and eosin staining (Left: Magnification × 100, Right: Magnification × 400). (H) Immunohistochemical CD68 (red), CD69 (green) staining of macrophages and T lymphocytes (Left: Magnification × 50, Right: Magnification × 200, *: PVA particles). (I) SEM image of PVA filling agent before implantation (Bar = 200 μm). (J) Diameter analysis of the PVA microspheres before and after filling.

**FIGURE 2:**
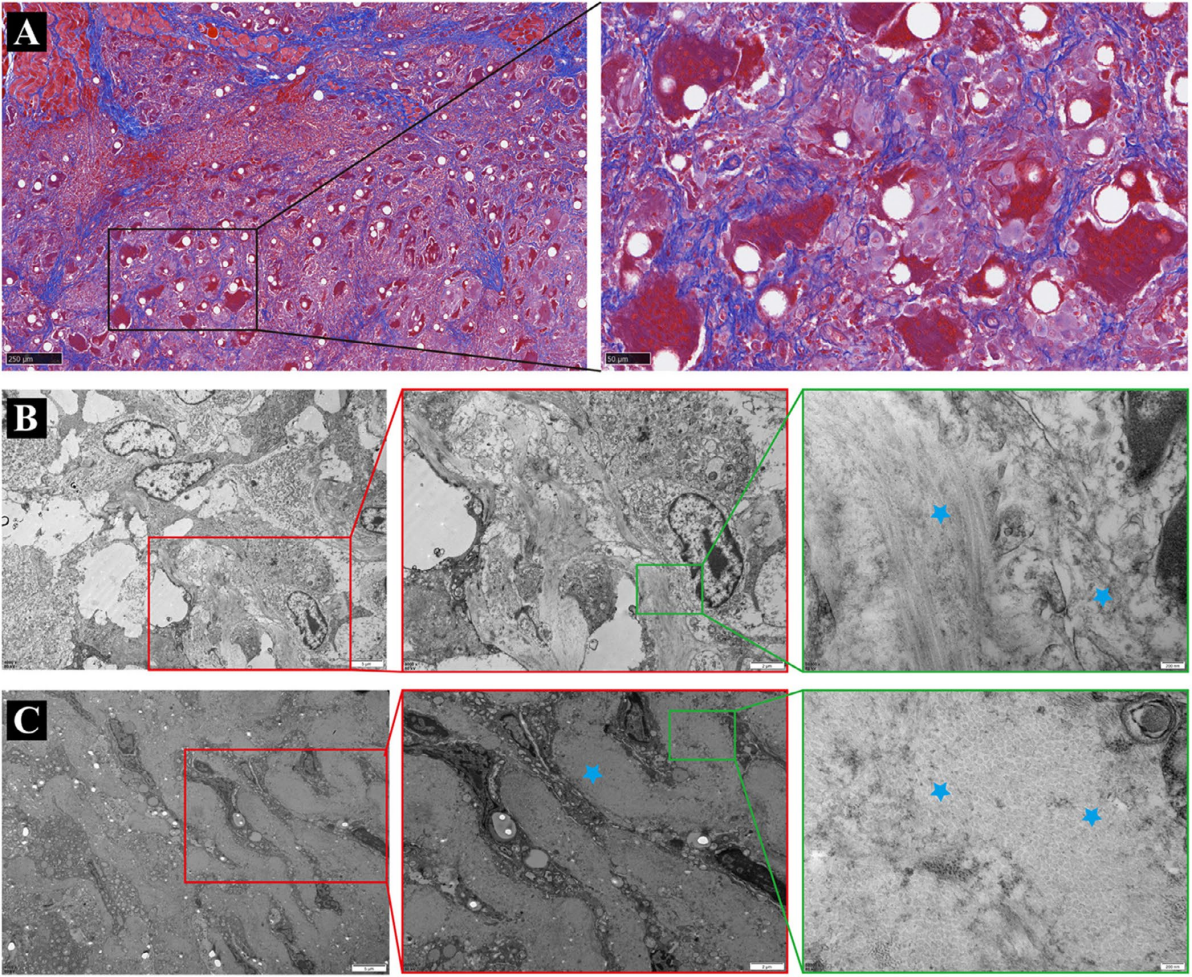
(A) Masson staining (Left: Magnification × 50, Right: Magnification × 200). (B) Transverse images of collagen fibers at different TEM magnifications (Left: Bar = 5 μm; Medium: Bar = 2 μm; Right: Bar = 200 nm). (C) Vertical images of collagen fibers at different TEM magnifications (Left: Bar = 5 μm; Medium: Bar = 2 μm; Right: Bar = 200 nm).

An ultrasound examination of the nodule revealed a hypoechoic deposit with an ill‐defined boundary, irregular shape, and a few dots of vascular signal in the subcutaneous tissue (Figure 1A,B). CT images showed slightly hyperdense lesions with ill‐defined boundaries and surrounding fibrosis (Figure 1C,D). During surgery, we found that the masses mixed with pale yellow substance were tightly adhered to the surrounding tissue (Figure 1E,F). Hematoxylin and eosin (HE) examination of the mass all demonstrated mild inflammation with infiltration of histiocytes and multinucleated giant cells around translucent nonbirefringent foreign PVA particles (asterisk) of defined shape (Figure 1G). Reactive asteroid body (Green arrow) in giant macrophages was also observed around PVA particles, further suggesting that the lesion may be undergoing a chronic granulomatous reaction (Figure 1G). Immunofluorescence staining revealed numerous CD68^+^ macrophages and few CD69^+^ T lymphocytes soaking and encasing the injected PVA materials (Figure 1H). The particle size distribution of PVA within the granulomas (29.79 ± 4.49 μm) was comparable to the electron microscopy (SEM) results of the filler (30.31 ± 5.59 μm) (Figure 1I,J), without showing signs of disintegration within a strong FBGs. Masson staining showed the cicatricial fibrosis invading surrounding tissues with fingerlike fibrous septa (Figure 2A). And the collagen fibers (blue asterisk) displayed disorganization and irregularity in shape in subsequent Transmission electron microscope (TEM) images (Figure 2B,C). Consistent with the reported literature, the diagnosis of the cases aligned more with the hallmark histological features of FBG [1].

## 2 Discussion

PVA is a water‐soluble, versatile synthetic polymer obtained by the alcoholysis, hydrolysis, or ammonolysis of polyvinyl acetate, which has a wide range of industrial, pharmaceutical, and biomedical applications [2]. The unique ability to form hydrogels and excellent characteristics of PVA, such as good biocompatibility, non‐toxicity, and excellent chemical stability, make it suitable for volume restoration in aesthetic and reconstructive fields [3]. As an injectable cosmetic filler, PVA material often needs to be formulated into microspheres and suspended in cellulose or sodium hyaluronate medium. The product of Bonita is a long‐acting filler containing PVA microspheres (30 mg/mL) and crosslinked sodium hyaluronate gel (10 mg/mL). It is often used as a volumizing agent filling the space between the deep dermis and the subcutaneous layer of the skin. The carrier is gradually absorbed over time, while the PVA microspheres hope to serve as the long‐term volume holder and even the stimulants for neocollagenesis. These PVA particles are typically larger than 15 μm (25–45 μm), making them difficult to phagocytose by a single cell. Ideally, PVA microspheres act as the stabilizers of filler and are kept by macrophages in a latent stage. This is often presented as a monolayer of macrophages or fibrocytes surrounding microspheres and enveloped by a zone of new fibrous tissue, which is the true histologic basis of the volume restoration and not a fibrosis reaction. Once some hazardous events are triggered, macrophages can be reactivated, resulting in the accumulation of foreign body giant cells to carry out their degradation.

Considering the absence of CD69‐positive immune cells (a marker for T‐cells), we are more inclined to attribute this adverse event primarily to a non‐adaptive immune response, rather than an adaptive immune reaction [4]. As noted by Alijotas‐Reig J, filler itself or its degradation products are more likely to act as adjuvants rather than T‐cell activators, causing inflammatory adverse events in a non‐specific manner [5]. This also explains why some inflammatory adverse events typically respond poorly to conventional treatment protocols. Therefore, clarifying the relationship between the causative factors and the immune response may aid in managing these adverse events. Notably, the timing of the appearance of T cells in cases may also be related to the implantation time or the filling area. Unfortunately, we are unable to identify potential triggers and specific pathogenesis that may be associated with the observed granulomatous reaction. The preoperative treatment measures taken for these three patients also remained unknown in detail.

Microsphere‐based fillers exhibit some specific histopathological characteristics, and understanding these features is valuable for identifying and diagnosing granulomatous reactions when clinical information is lacking. The histological characteristics of FBG induced by PVA‐based fillers are generally distinguishable from those caused by fillers like calcium hydroxyapatite (CaHA), poly‐L‐lactic acid (PLLA), and dextran microspheres. For example, crackled particles of bluish‐gray staining are often consistent with CaHA, while PLLA particles exhibit birefringence. Dextran beads may present with a blue‐stained appearance. This is especially significant when patients intentionally withhold or are unaware of the medical history information related to the association between injectable fillers and symptoms. Of note, the FBG reactions due to both permanent PVA and PMMA injections are sometimes similar and challenging to distinguish from histopathological findings. Meanwhile, the FBG response of PVA demonstrated in this study is not a unique histological manifestation of PVA material. Considerably more work is needed to be done to elucidate the histological presentation and mechanism of PVA‐induced FBG.

As latent “living implants,” the semi‐permanent property of PVA particles lack effective solvents and can exert long‐term stimulants for granumatous reaction, leading to the FBGs that are intractable, persistent and recurrent. Therefore, the timing and progress of management actions are fundamental to the outcomes of resolve, remission, and exacerbation. Firstly, it is crucial to distinguish early nodule‐like reactions after the injection of microsphere‐based fillers, which could be the result of particle accumulation rather than a granulomatous reaction. As for some later‐stage FBGs, being managed with intralesional steroid, 5‐fluorourail, methotrexate, bleomycin, and prednisone injections, or combined with oral anti‐inflammatory agents and energy‐based devices have been typically suggested as the symptomatic treatments [6]. Appropriate tissue dispersing agents such as collagenase or hyaluronidase can be selected for long‐standing FBGs with fibrous encapsulation. Unfortunately, there is currently no research findings available on antidotes for PVA filler. Surgical intervention is usually the final useful option for dealing with some intractable or permanent granulomas. Due to the infiltrative nature of granulomas, it is difficult to completely remove lesions through surgery. This is also the reason why postoperative discomfort persisted or lesions recurred in some cases.

Conclusively, some insights gained from this study may be of assistance for clinicians to be aware and prevent this unfavorable adverse event of PVA injection. Long‐term follow‐up studies of filler injection as well as postprocedural outcomes need to examine more closely the links between safety and efficacy.

## Author Contributions

**Hang Wang:** conceptualization, project administration, data curation, formal analysis, investigation, writing – original draft. **Chihchieh Lo:** investigation, data curation. **Dongmei Wu:** formal analysis, investigation, visualization, supervision. **He Qiu:** conceptualization, data curation, formal analysis, investigation, writing – original draft.

## Ethics Statement

Authors declare that human ethics approval was not needed for this study.

## Conflicts of Interest

The authors declare no conflicts of interest.

## Data Availability

The data that support the findings of this study are available from the corresponding author upon reasonable request.
